# An Evidence-Based Rapid Review of Surgical Techniques for Correction of Prolapsed Nictitans Glands in Dogs

**DOI:** 10.3390/vetsci5030075

**Published:** 2018-08-23

**Authors:** Constance White, Marnie L. Brennan

**Affiliations:** 1 Fremont Veterinary Clinic, Portland, OR 97213, USA; doctornev2000@yahoo.com; 2 Centre for Evidence-based Veterinary Medicine, School of Veterinary Medicine and Science, University of Nottingham, Nottingham LE12 5RD, UK

**Keywords:** eye, ophthalmology, prolapsed nictitans gland, prolapsed third eyelid, cherry eye, canine, dog, veterinary surgery, rapid review

## Abstract

Prolapsed nictitans gland (PNG) is an important ocular condition of dogs. Various surgical interventions have been described, but effective technique is currently considered to be a matter of personal clinician preference. The aim of this rapid review was to evaluate existing peer-reviewed evidence of effectiveness for surgical techniques and their subsequent effects on quantitative and clinical lacrimal outcomes for PNG. We performed a structured bibliographic search of CAB Abstracts, PubMed, and Medline using terms relevant to dogs, nictitans gland, and surgery on 13 September 2017. Included studies were assessed for study design, reporting characteristics, surgical techniques, and surgical and lacrimal outcomes. Fifteen of three hundred fifteen identified studies were eligible for inclusion. Seven different replacement techniques were identified, along with gland excision. All studies were observational or descriptive, with the exception of a single crossover trial. Outcomes reporting was heterogeneous and provided limited detail on lacrimal outcomes or on breed propensity for recurrence. Insufficient data precluded comparison of techniques for either surgical failure rates or lacrimal outcomes, although proportional meta-analysis yielded an overall failure rate of 3% (95% CI 1–7%) for the Morgan’s pocket procedure. Improved reporting of veterinary surgical studies will improve evidence appraisal and synthesis, as well as reduce potential sources of bias.

## 1. Introduction

The third eyelid or nictitans membrane is a conjunctival fold in the ventromedial fornix of many mammalian eyes [1] (p. 961). In the dog, it is supported by a T-shaped cartilage and contains the nictitans gland, which contributes to the aqueous portion of tear film [2]. A prolapsed nictitans gland (PNG) is thought to be one of the most common disorders affecting the canine ocular adnexa [3] (p. 79). Laxity of connective tissue attachment between the gland and ventral periorbital tissues, as well as antigen-stimulated gland enlargement, are thought to be predisposing risk factors [1] (p. 963) [4] (p. 1161). Incidence of prolapse varies by breed and is thought to have a genetic contribution [5,6,7] (pp. 442–452). Prolapse is most often seen in juvenile animals, with the preponderance of cases occurring in animals under two years of age [1] (p. 963).

Although gland excision was formerly the mainstay of treatment [8], removal is now discouraged due to concerns for subsequent development of dry eye (keratoconjunctivitis sicca; KCS). Removal of the gland may decrease Schirmer tear test (STT) values, although the relative decrease reported is highly variable [2,9,10]. Currently, replacement is recommended, with choice of surgical technique suggested to be a matter of personal clinician preference [1] (p. 964) [11], since no systematic comparisons are available for surgical outcomes. Similarly, different techniques are thought to vary in efficacy due to surgical complexity, breed conformation differences, effects on third eyelid mobility and cartilage position, gland function, and risks of complications and surgical failure [12,13,14]. In particular, repair is thought to be less successful in some giant and brachycephalic breeds (e.g., Neapolitan Mastiffs, English Bulldogs) [15] (p. 209) [16] (p. 54) and in cases of prolapse of long standing duration [17]. Finally, topical corticosteroid use remains controversial; some veterinarians suggest that risk of surgical failure is higher in hypertrophied or inflamed glands while others discourage the use of perioperative steroids [18,19]. Currently, one technique (Morgan Pocket) is favored by a number of veterinarians, as it is thought to be easy to master and fairly effective [1] (p. 964) [3] (p. 80) [12].

The aim of this study was to systematically search the veterinary literature to identify and evaluate surgical techniques reported in peer-reviewed publications for the treatment of PNG. Specifically, we sought to examine rates of surgical failure (reprolapse), lacrimal outcomes (quantitative measures and development of KCS), and frequency of surgical complications, as well as design and reporting characteristics of included studies. Additionally, we sought to assess for prognostic effect of breed, perioperative treatment, and duration of prolapse on these outcomes.

## 2. Materials and Methods

Ethical approval for the study was received from the ethics committee at the School of Veterinary Medicine and Science at The University of Nottingham (approval number 2059 170706).

### 2.1. Search Strategy

A search of CAB Abstracts (1910–present), Medline (In-Process & Other Non-Indexed Citations and MEDLINE(R) 1946 to Present), and PubMed (1948–2016) was performed in February 2016 and was updated 13 September 2017 using the OVID interface for CAB Abstracts and Medline, and the native PubMed interface. The abstract, title, broad terms, and heading words were searched (keyword search) using terms relevant to dogs (canine, canines, canid, canids, canis, canidae, dog, dogs), nictitans gland or membrane (nictitans, nictitans, third eyelid, third eyelids, cherry eye, cherry eyes, nictitating membrane, nictitating membranes), and prolapse or surgery (prolapse, prolapsed, prolapses, replacement, replacements, replaced, replace, surgery, surgical, repair, amputate, amputated). The searches also employed medical subject heading searching and were linked with Boolean terms as described in the supplement (Table S1 in Supplementary File).

### 2.2. Inclusion Criteria

The inclusion criteria for publications were as follows: (1) relate to the treatment of naturally occurring PNG in dogs; (2) study design must be a case series (report of >1 case), case control, cohort, or controlled or uncontrolled trials; (3) be written in the English language; (4) be primary research; (5) be in a peer-reviewed journal accessible by the authors via the University of Nottingham or the British Library.

### 2.3. Exclusion Criteria

Exclusion criteria were as follows: (1) unrelated to PNG condition or surgical intervention (not PICO); (2) non-English language reports; (3) be an editorial, instructional review, conference abstract, book chapter, or experimental report; (4) not published in peer-reviewed journals; (5) single case report; (6) full text unavailable to the authors via the University of Nottingham library or the British Library.

### 2.4. Application of Inclusion and Exclusion Criteria

Initial screening by title and abstract was applied by a single author (CW) and a random sample of 20% of publications was independently appraised by the second author (MB) to ensure fidelity of inclusion/exclusion criteria. After excluding those that did not meet the inclusion criteria, articles of interest were reviewed in detail (full text) by both the authors and a decision was made regarding inclusion into the study.

### 2.5. Data Extraction

Study type, procedural details, patient demographics, number of patients operated on and number of eyes, study and follow-up periods, failure and complication rates, and lacrimal function outcomes were extracted systematically using a template piloted (Table S2 in Supplementary File) by a single author (CW). Data extracted from included studies was verified by the second author (MB). In cases of disagreement between both authors with regard to study inclusion or data extraction, a third reviewer was used for the final decision. Reporting characteristics were assessed using criteria detailed in Table S2. Meta-analysis of lacrimal outcomes (STT outcomes, KCS incidence) and surgical failure rates overall, as well as potential effect modification by breed, perioperative treatment, or duration of prolapse, were considered in the initial review protocol.

### 2.6. Study Design

Classification of observational and descriptive studies is currently an area of considerable debate [20,21]. Descriptive studies reporting outcomes from a single intervention were considered to be surgical case series [21,22,23]. Observational studies that compared the treatment effects of two or more interventions were considered to have features of a cohort study [20,21,22,24].

### 2.7. Statistical Analysis

Data analysis was performed using Stata IC13. Proportions were calculated for percentage recurrence when not provided in the papers. To perform proportional meta-analysis of surgical failure rates, we used the Metaprop statistical procedure to pool prevalence based on the Wilson score test statistic using a random effects model [25]. Use of the Freeman-Tukey double arcsine transformation to stabilize the variances allows for improved summary effect estimates when proportions are close to 0 or 1 [26]. Wilson score confidence intervals were calculated for individual studies. Confidence intervals were calculated per operated eye, with the exception of two publications that reported only recurrence per patient (for those reports, we assumed unilateral prolapse for conservative estimates of confidence intervals). Schirmer tear test results extracted from raw data were checked for normality and paired *t* testing was used to assess for mean difference found for pre- and post-operative eyes. Statistical significance was set at *p* < 0.05.

## 3. Results

**Figure 1:**
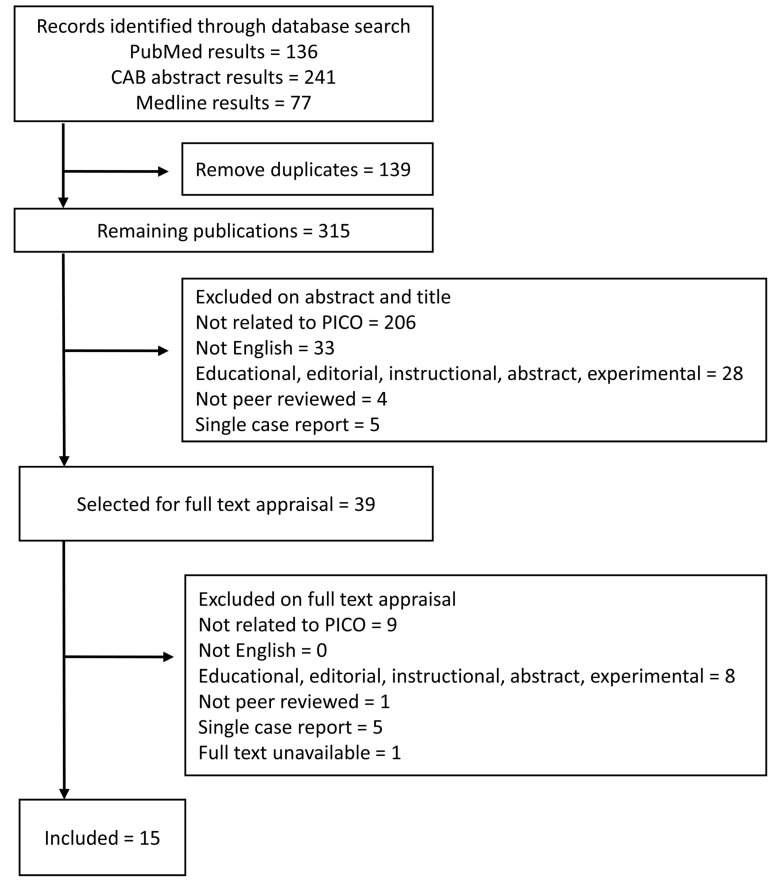
A summary of the PNG rapid review process.

The initial search yielded 315 unique publications of which 39 met inclusion criteria sufficiently to warrant full text evaluation (Figure 1). Of those 39, 16 publications were identified for inclusion; only 15 of those were available for full text appraisal. Third party review was not required, since there were no disagreements regarding inclusion between the two authors.

### 3.1. Techniques Identified

**Figure 2:**
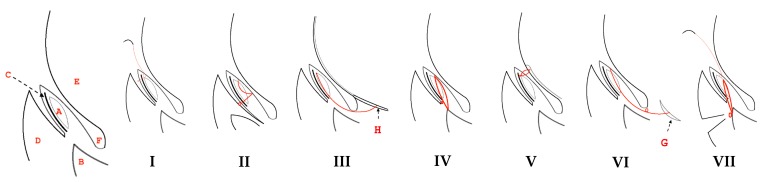
Schematic representation of replacement techniques identified during a rapid review of the literature on PNG. (**I**) Morgan pocket; (**II**) Intranictitans tack; (**III**) Inferior scleral anchor; (**IV**) Periosteal anchor; (**V**) Perilimbal pocket; (**VI**) Ventral rectus anchor; (**VII**) Periosteal anchor combined with Morgan pocket. Surgical anatomy: A. third eyelid gland; B. ventral orbit; C. third eyelid cartilage; D. inferior palpebra; E. globe F. fornix; H. inferior sclera; G. ventral rectus; —suture o knot.

Eight surgical techniques comprising seven replacement procedures, along with gland excision, were identified in the 15 included publications (Figure 2, Table 1). Replacement procedures could be divided into those fixing the gland to the third eyelid itself, without alteration of third eyelid mobility, or to other ocular or adnexal tissues with concomitant restriction of mobility.

#### 3.1.1. Techniques Involving Fixing the Gland to the Third Eyelid

Morgan, Duddy, and McClurg [27] first reported a technique burying the gland into a pocket created on the bulbar surface of the third eyelid using absorbable suture (“Morgan pocket”, Figure 2I). Use of this technique was subsequently described in seven additional publications that met the inclusion criteria [17,28,29,30,31,32,33]. Plummer and colleagues [14] described an alternative technique in which the gland is tacked to the cartilage of the third eyelid using non-absorbable monofilament suture (“intranictitans tack”, Figure 2II).

#### 3.1.2. Techniques Involving Fixing the Gland to Other Ocular or Adnexal Structures

One study [27] used a previously described technique [34,35] tacking the gland to ventral sclera (“inferior scleral anchor”) (Figure 2III). Kaswan and Martin [36] first described suture fixation of the gland to the ventral orbital periosteum (“periosteal anchor”) (Figure 2IV); this technique was used in two additional included articles [29,37]. Premont and others [38] reported a technique that buries the gland in ventral episcleral tissues via a 160° perilimbal incision (“perilimbal pocket”), with a temporary tarsorrhaphy maintained for two weeks (Figure 2V). Sapienza, Mayordomo, and Beyer [11] described a technique in which the gland is anchored to the ventral rectus ocular muscle (“ventral rectus anchor”, Figure 2VI). Suture material used in these techniques were non-absorbable, with the exception of the perilimbal pocket procedure.

#### 3.1.3. Combination of Techniques

Multari and coworkers [32] combined the Morgan pocket technique with a periosteal anchor placed via a palpebral approach. Pocketing was modified slightly by use of a small conjunctivectomy over the gland (Figure 2VII).

#### 3.1.4. Excision

Five publications were identified in which gland excision was either the sole treatment or one of the treatments evaluated [27,29,37,39,40]. Few surgical details other than those regarding hemostasis were included in those publications.

### 3.2. Study Characteristics

#### 3.2.1. Design and Sampling

All but one publication were observational or descriptive studies (Table 1). Most described results used a single surgical technique whilst four described cases using ≥2 techniques. Patient (case) source and selection methodology was often incompletely reported (Table 1). Five reports (four identified as retrospective chart reviews [11,27,31,32]) described periods of enrollment and study locations. Seven reports did not specify whether data collection was prospective or retrospective, or provide the time period and/or locations from which patients were accrued [17,29,30,33,36,39,40]. The two prospective studies gave few details on patient recruitment or eligibility criteria [14,38].

#### 3.2.2. Reporting

Follow-up intervals, when reported, varied widely both within and between studies, ranging from two weeks to 10 years, with two studies using a minimum follow-up period as inclusion criteria [11,31] (Table 2). Follow-up intervals were variably described using minimums [11,17,31], means [27], ranges [27,28], as patient-level raw data [14,38], as a single interval [30,33], was unclear [29], or not reported [32,36,39,40]. When attrition was documented, characteristics of those lost to follow-up were not described [27].

Surgical failure (reprolapse) after replacement was reported in nearly all studies (Table 2). However, breed-specific failure rates were reported completely only in the two studies that provided patient-level data [14,38]; two others reported outcomes for selected breeds [32] or enumerated recurrence events by breed without denominator [31]. Schirmer tear test (STT-1) outcomes were described in four studies [14,29,37,38]; however, timing of post-operative STT-1 was uncertain and/or variable in two [14,38] and over a relatively short follow-up period in the remainder [29,37]. KCS incidence in all or a subset of included patients was described in five reports [11,27,29,31,38], although only one study reported KCS in a group followed over a relatively long term (minimum of two years) [27]. Post-operative complications (apart from failure) were usually reported, though often in narrative form. Duration of prolapse prior to surgery was described in just over half of the included studies although, apart from the two reports containing patient-level data, was usually given as a range with a measure of central tendency (mean or median). Apart from a single study that analyzed post-operative STT-1 values with adjustment of breed and prolapse duration [38], no other reports analyzed outcome data with stratification or adjustment for these putative prognostic factors.

### 3.3. Study Outcomes

Eight studies reported outcomes of the Morgan pocket technique whilst three studies described results with periosteal anchoring; other techniques were each represented by a single publication (Table 3). Most studies had a small sample size, generating wide calculated confidence intervals for surgical failure (reprolapse). Variations in perioperative care and surgical technique were found for procedures with more than one report: Morgan pocket procedures varied in suture material, use of conjunctivectomy, use of perioperative corticosteroids, and layers of closure whilst periosteal anchor procedures varied in suture choice. Frequency and management of cartilage deformities were not uniformly reported.

#### 3.3.1. Surgical Failure

Reprolapse of the gland occurred in 0–58.9% of cases reported (Table 3). Incidence of surgical failures ranged from 0–12.5% for Morgan pocket procedures, from 0–25% for periosteal anchor alone, and from 0–9% for all other procedures excepting inferior scleral anchoring, which suffered a much higher failure rate (58.9%). No failures were reported using ventral rectus anchoring.

**Figure 3:**
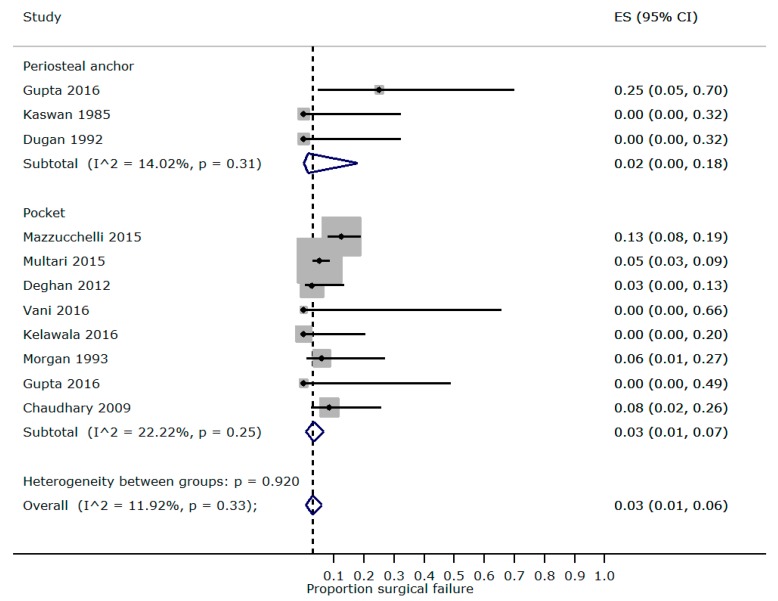
Surgical failure after replacement surgery from included studies from a rapid review of surgical interventions for PNG with proportional meta-analysis of publications reporting outcomes of the Kaswan periosteal anchor and Morgan pocket techniques; CI = Wilson score 95% confidence interval; ES = effect size (proportion surgical failure); I^2^ = Higgins’s heterogeneity statistic.

We were able to perform proportional meta-analysis for studies reporting outcomes from Morgan pocketing and periosteal anchoring techniques (Figure 3) but without stratification for breed, perioperative steroid, and prolapse duration as originally planned (due to incomplete reporting). Mild heterogeneity (Morgan I^2^ = 22.2%, periosteal anchor I^2^ = 14.0%) was found between with studies of each technique, with a pooled recurrence rate of 3% (95% CI 1–7%) for Morgan pocketing and 2% (95% CI 0–18%) for the periosteal anchor.

Breed-specific recurrence rates, particularly with reference to brachycephalic and giant breeds, were not available in sufficient detail from most studies to allow stratified analysis. However, one study [32] reported a higher incidence of recurrence in Bulldogs using the Morgan pocket technique (11.9%, 95% CI 5.2–25.0%) rather than the pocket combined with a periosteal anchor (3.2%, 95% CI 0.9–11.0%). In that study, surgical technique had not been assigned randomly but selected on the basis of predicted surgical failure.

#### 3.3.2. Complications

Postoperative complications were discussed in seven studies but were not systematically reported. The most common reported complication was corneal ulceration (Morgan pocket 6/440 eyes, Morgan pocket with periosteal tack 5/186, perilimbal pocket 2/44 eyes). Third eyelid elevation or gland prominence was reported in 2/234 Morgan pocket eyes, 3/20 periosteal anchor eyes, and 8/186 eyes in which the Morgan pocket was combined with periosteal anchoring. One lacrimal cyst was reported (Morgan pocket). Postoperative cartilage eversion was reported in two cases using the Morgan pocket technique and in two eyes using the intranictitans tack.

#### 3.3.3. Lacrimal Outcomes

Time points and data analysis of STT-1 was unique to each of the three studies that reported quantitative lacrimal outcomes (Table 4). Dugan and others [37] analyzed STT-1 over time using repeat measures ANOVA, Plummer and colleagues [14] reported raw data, while Prémont et al. [38] presented raw data but used mixed modelling to estimate pre- and postoperative STT-1 least square means (with adjustment for breed, prolapse duration, contralateral eye correlation, and time).

In dogs followed for up to 24 weeks after gland excision, mean STT-1 was reduced by a small but statistically significant amount as compared to the unaffected eye [37]; a smaller and statistically insignificant decrease was seen in eyes that underwent periosteal tacking followed for up to 16 weeks [37] (Table 4). Replacement using either intranictitans tacking or perilimbal pocketing resulted in increases in measured mean STT-1 [14,38], although differences were significant only in the mixed model least squares mean for the latter.

Quantitative comparison of KCS outcomes per procedure was not possible due to the small number of studies reporting KCS incidence and varying follow-up. Two case series reported no incident KCS following replacement by ventral rectus anchor or perilimbal pocket [11,38]. A third descriptive study reported three new cases of KCS in 144 eyes that underwent Morgan pocketing [31].

A cohort study [27] that followed 66 eyes over ≥2 years reported higher risk (RR, relative risk 3.4) of KCS in eyes in which the gland was excised (13/27 eyes) rather than replaced with inferior scleral anchor or Morgan pocket techniques (2/14 eyes). Additionally, untreated prolapse also increased relative risk of KCS (RR 3.0) as compared to replacement in this study, although statistical significance was not achieved (possibly due to small sample of untreated glands, n = 7); we were unable to identify any other publications describing lacrimal outcomes for chronic untreated prolapse. However, differential loss to follow-up occurred between treatment arms (55.7% excised, 36.4% untreated, 68.2% replaced), and differences in time-at-risk were not fully adjusted for in the analysis. A second study reported one case of KCS in eight eyes treated by gland excision over three years of follow-up [29].

## 4. Discussion

To our knowledge, this is the first structured evidence-based review of surgical therapies for PNG in the dog. Fifteen studies were identified encompassing seven different techniques for gland replacement, along with excision, in 809 dogs and approximately 1019 eyes. Surgical failure rates were comparable between all replacement techniques, with the exception of the inferior scleral anchor, which appeared to suffer from more frequent failure. Only Morgan pocketing and Kaswan periosteal anchoring were reported in a sufficient number of studies with adequate detail to allow estimation of a summary surgical failure rate.

Breed, prolapse duration, and perioperative steroid treatment are suggested to be important prognostic factors for PNG replacement success [4,15,17,18,19]. We attempted to estimate whether different techniques varied in failure rates for breeds thought to be more prone to reprolapse (Mastiff breeds, Shar-Pei, Newfoundland, Bulldog) [1,4,15,16,19]. While included publications generally reported breed composition of study participants, outcome data was less granular and did not allow for stratification of failure rates by breed. We suspect that such analysis might have yielded lower success rates for some breeds, given a replacement surgery failure rate of 39.8% reported in an unpublished survey of Neapolitan Mastiff owners in the United Kingdom (K. South, United Kingdom Neapolitan Mastiff Club, personal communication). Similarly, incomplete reporting of prolapse duration and perioperative care precluded analysis of the effect of these factors on surgical outcomes. We hypothesize that prolonged prolapse might yield higher failure rates if such an analysis were available. Contrariwise, preoperative ocular steroids may increase surgical success of all techniques by decreasing inflammatory gland hypertrophy whilst post-operative ocular steroids could delay incisional healing, resulting in higher failure rates in techniques reliant on imbrication for stable replacement.

In addition to heterogeneous perioperative medical care, small but potentially important differences in technique were also found for procedures with more than a single publication. Two layer closure has been suggested to improve the surgical success of Morgan’s pocket technique (R.V. Morgan, personal communication), but not all ophthalmologists agree with this suggestion [1,18]. Insufficient data precluded comparison of single versus double layer closure. Likewise, different suture materials used in studies reporting the Morgan pocket technique vary in biomechanical properties [41,42], which could potentially affect surgical outcomes. Finally, details of cartilage excision were also incompletely reported in most studies; cartilage scrolling or eversion may accompany PNG and is thought to affect surgical success [19].

Effects on lacrimal function were not routinely reported and this prevented estimates or comparison of lacrimal outcomes for the reported techniques. The finding of a significant, though small, decrease in STT-1 after gland excision [37] is concordant with other studies examining the effect of gland removal in normal dogs and dogs with prolapse over periods of up to one year [2,9,43], although is at odds with prior work suggesting reduction of up to 57% [10]. Postoperative KCS was not routinely reported in most studies. Follow-up duration may not have been adequate for capture of true KCS incidence, given the reported delay of a median of 4.5 years between prolapse/intervention and subsequent KCS [27]. We note that the association of KCS with gland excision relies heavily on a single cohort study [27] with differential loss to follow-up between exposure groups and without time-to-event analysis. Although the recognition of gland excision as a possible risk factor for KCS is quite important, more robust analysis with extensive follow-up would be required to definitively confirm this association, given the long latency of KCS [44,45].

Most included studies were at high risk for bias: sampling methods were frequently not reported, increasing the risk of selective reporting [46]. In studies assessing multiple interventions, intervention assignment was non-randomized, making comparison between groups difficult [47]. Characteristics of those lost to follow-up were not detailed, increasing risk of possible attrition bias [48]. Studies that used a minimum follow-up time for inclusion are at higher risk of having missed data bias due to patients lost prior to the minimum follow-up period potentially being systematically different from patients included in the study [48,49].

Observational and descriptive studies comprise much of the clinical evidence in veterinary medicine [50,51,52,53]. Inadequate reporting has been associated with biased outcome measures in human medicine [54] and is suggested to increase risk of bias in veterinary studies [55]. A clear definition of study design and aims, sources of and selection criteria for patients, complete accounting of loss to follow-up and missing data, along with standardized a priori definitions and measurement of explanatory and outcome variables are suggested to minimize bias and improve evidentiary value [22,55,56]. Additionally, long latency outcomes are better assessed using time-to-event statistical methods [48]; conversion of continuous data such as STT-1 values into clinically meaningful categorical data would allow for similar statistical handling. It is not our intention to suggest specific guidelines for the reporting of veterinary ophthalmology studies, but study quality may be improved by use of the STROBE-Vet reporting guidelines for cohort studies [57] and newly developed guidelines for the reporting of veterinary case series [22]. For reports of novel surgical techniques, use of the PROCESS reporting guidelines [23], particularly with regard to modifications, complications, operator experience, and learning curves, may help in the assessment of utility and feasibility by other surgeons. Finally, open sharing of raw data eases data extraction for meta-analysis, allows re-analysis by others with secondary hypotheses, and may reduce statistical inconsistencies and errors [58,59]. Additionally, patient-level data may be combined from multiple centers to assess prognostic/baseline factor effects on treatment success with greater statistical power [60]. We encourage authors of surgical case series or cohort studies to share anonymized patient-level clinical data through a data sharing plan or as supplementary materials in a publication.

This review had a number of limitations. We searched databases shown to provide the most comprehensive coverage of the veterinary literature but excluded foreign language and non-peer reviewed publications, as well as grey literature such as textbooks, websites, and other commonly accessed forms of evidence. Inclusion of non-English language reports may have altered the pooled estimate for the Morgan pocket surgery success [61,62,63,64]. We are also aware that at least two non-English studies assess multiple techniques [65,66], as well as a microsurgical technique different from those captured by this review published in a French language journal [67].

## 5. Conclusions

The procedures found in our rapid review vary substantially in technical difficulty, as well as in potential complications. Although the clinician may choose from a variety of techniques described for gland replacement, there is currently insufficient evidence to ascribe superior surgical success rates and lacrimal outcomes to any particular technique. We note in particular that gland excision may predispose to development of KCS, but further studies are needed to confirm this association and the effect of breed on subsequent development of KCS after excision or replacement. Quality of reporting can be improved in observational and descriptive studies to reduce potential bias. Most studies were generated from referral populations under the care of veterinary ophthalmic specialists. Generalization of reported results to primary care populations may not be appropriate, particularly given potential differences in patients and operator experience.

## Figures and Tables

**Table 1:** Included studies, procedures, study designs, and whether patient sources were stated in papers identified during a rapid review of surgical interventions for PNG. R = reported (white); PR = partially reported (light gray); NR = not reported (dark gray); NA = not applicable (white).

Publication	Procedure(s)	Study Design	Patient Source and Selection Method
Arora et al., 2014 [39]	Gland excision	Case series	NR
Chaudery et al., 2009 [28]	Morgan pocket	Case series	R
Dehghan et al., 2012 [17]	Morgan pocket	Case series	NR
Dugan et al., 1992 [37]	1. Periosteal anchor 2. Gland excision	Crossover trial	NA
Gupta et al., 2016 [29]	1. Gland excision 2. Periosteal anchor 3. Morgan pocket	Case series	NR
Kaswan and Martin, 1985 [36]	Periosteal anchor	Case series	NR
Kelawala et al., 2016 [30]	Morgan pocket	Case series	NR
Mazzucchelli et al., 2012 [31]	Morgan pocket	Retrospective case series/prevalence study	R
Morgan et al., 1993 [27]	1. Inferior scleral anchor 2. Gland excision 3. Morgan pocket	Retrospective case series with cohort analysis	R
Multari et al., 2016 [32]	1. Morgan pocket 2. Morgan pocket combined with periosteal anchor	Retrospective case series with cohort analysis	R
Plummer et al., 2008 [14]	Intranictitans tack	Prospective case series	NR
Prémont et al., 2012 [38]	Perilimbal pocket	Prospective case series	PR
Sapienza et al., 2014 [11]	Ventral rectus anchor	Retrospective case series	R
Sarma, 2010 [40]	Gland excision	Case series	NR
Vani and Lakshmi, 2016 [33]	Morgan pocket	Case series	NR

**Table 2:** Reporting of explanatory and outcome variables in studies identified during a rapid review of surgical interventions for prolapsed nictitans gland in the dog. STT = Schirmer Tear Test; KCS = keratoconjunctivitis sicca; R = reported (white); PR = partially reported (light gray); NR = not reported (dark gray); NA = not applicable (white).

Publication	Follow-Up Time	Surgical Failure (Reprolapse)	Surgical Failure (Reprolapse) by Breed	STT	KCS
Arora et al. [39]	NR	NA	NA	NR	NR
Chaudery et al. [28]	R (3–4 months)	PR ^4^	NR	NR	NR
Dehghan et al. [17]	R (≥6 months)	R	NR	NR	R
Dugan et al. [37]	R (24 weeks)	R	NA ^6^	R	NR
Gupta et al. [29]	PR (≤3 years)	Unclear ^5^	NR	PR ^10^	R
Kaswan and Martin [36]	NR	PR ^4^	NR	NR	NR
Kelawala et al. [30]	R (1 month)	R	NR	NR	NR
Mazzucchelli et al. [31]	R (≥1 year ^1^)	R	PR ^7^	NR	R
Morgan et al. [27]	PR (33/89 ≥ 2 years)	R	NR	NR	PR ^11^
Multari et al. [32]	NR	R	PR ^8^	NR	NR
Plummer et al. [14]	R (0.5–33 months ^2^)	R	R	R	NR
Prémont et al. [38]	R (2–62 months ^3^)	R	R	R	R
Sapienza et al. [11]	R (≥1 year ^1^)	R	NA ^9^	NR	R
Sarma [40]	NR	NA	NA	NR	NR
Vani and Lakshmi [33]	R (6 months)	R	NA ^9^	NR	NR

^1^ Inclusion criteria; ^2^ Calculated median 21.5 months, STT follow-up NR; ^3^ Calculated median 21.5 months, STT/KCS median 5.5 months; ^4^ Reported per patient rather than per eye; ^5^ One case reported as “partial recovery”; ^6^ Single breed trial; ^7^ Breeds enumerated but denominator unclear; ^8^ Selected breeds only; ^9^ No recurrence events; ^10^ Qualitative statement, no data; ^11^ Reported for 33/89 dogs.

**Table 3:** Procedural details and surgical failure rates reported by 13 publications describing results from PNG replacement surgery. NR = not reported.

Procedure	Publication	Preoperative Steroid	Postoperative Steroid	Surgery Notes	Operated Eyes	Reprolapse	Reprolapse (%)	95% CI ^1^
**Morgan pocket**	Chaudery et al. [28]	Yes	Yes	2-layer closure 5–0 Vicryl Cartilage excision NR	24	2 ^3^	8.3	2.3–25.8
	Dehghan et al. [17]	NR	No	2-layer closure 6–0 to 7–0 PDS or Vicryl Cartilage excision NR	38	1	2.6	0–13.5
	Gupta et al. [29]	NR	Yes	2-layer closure 6–0 Vicryl Cartilage excision NR	4	0	0	0–49.0
	Kelawala et al. [30]	Yes	Yes	1-layer closure 5–0 Vicryl Cartilage excision NR	15	0	0	0–20.4
	Mazzucchelli et al. [31]	NR	NR	NR	144	18	12.5	8.1–18.9
	Morgan et al. [27]	NR	NR	1- or 2-layer closure 5–0 Dexon or 6–0 Vicryl Cartilage excision NR	18	1 ^2^	5.9	1–27.0
	Multari et al. [32]	NR	No	Conjunctivectomy 1-layer closure 5–0 Monosyn Cartilage excised if everted	234	12	5.1	3.0–8.7
	Vani and Lakshmi [33]	NR	No	1-layer closure 3–0 catgut Cartilage excision NR	2 ^3^	0	0	0.0–65.8
**Periosteal anchor**	Dugan et al. [37]	NR	Yes	3–0 Ethilon	8	0	0	0–32.4
	Kaswan and Martin [36]	NR	Yes	3–0 monofilament	8^3^	0	0	0–32.4
	Gupta et al. [29]	NR	Yes	3–0 Prolene	4	1 ^4^	25.0	4.6–70.0
**Inferior scleral anchor**	Morgan et al. [27]	NR	NR	4–0 silk	51	30	58.9	45.2–71.2
**Morgan pocket with periosteal anchor**	Multari et al. [32]	NR	No	3–0 to 1 nylon for periosteum Cartilage excised if everted	186	9	4.8	2.6–8.9
**Intranictitans tack**	Plummer et al. [14]	Yes	Yes (6–8 weeks)	4–0 nylon Cartilage excised subsequently on one patient who developed eversion	15	1	6.7	1.2–29.8
**Perilimbal pocket**	Prémont et al. [38]	Yes in some	No	6–0 braided Vicryl Cartilage excised if everted	44	4	9.1	3.6–21.2
**Ventral rectus anchor**	Sapienza et al. [11]	NR	No	5–0 Ethilon Cartilage excised routinely	122	0	0.0	0–3.1

^1^ Calculated by authors using Wilson score confidence interval. ^2^ One lost to follow-up ^3^ Number of eyes not reported, number of dogs used as surrogate for number of eyes ^4^ One patient reported as “partial recovery”.

**Table 4:** Quantitative lacrimal outcomes reported in three studies from a rapid review of surgical interventions for PNG.

Publication	STT-1 Data	Procedure	Eyes	Follow-Up	Statistics Method	Preoperative STT-1 mm	Postoperative STT-1 mm	Mean Difference (mm)	*p* Value
Dugan et al. [37]	Mean difference ^1^	Excision (group 1)	3	15 weeks	Repeat measures ANOVA	NR	NR	−2.0	<0.01
		Excision (group 2)	3	24 weeks		NR	NR	−1.6	<0.001
		Excision (group 3)	3	15 weeks		NR	NR	−1.1	<0.01
		Periosteal anchor	6	8 weeks		NR	NR	−0.7	NR
		Periosteal anchor	3	16 weeks		NR	NR	−0.8	NR
Plummer et al. [14]	Raw data	Intranictitans tack	11	NR	None	18.5 ± 3.1 ^2^	21.2 ± 3.7 ^2^	2.7	0.1454 ^2^
Prémont et al. [38]	Least square means ^3^	Perilimbal pocket	19	5.5 months median range 0.5–48 mo.	Linear mixed model	19.6 ± 1.1	23.7 ± 1.1	4.1	0.003

Group 1 prolapsed 9 weeks then excised, Group 2 excised at time of prolapse, Group 3 excised 9 weeks after surgical replacement ^1^ Between affected and unaffected eyes on same individual ^2^ Pre- and postoperative STT-1 operated eyes calculated from raw data for operated eyes without recurrence and for which postoperative STT data was reported, paired *t* test.

## Supplementary Materials

The following are available online at http://www.mdpi.com/2306-7381/5/3/75/s1, Table S1: Search strategies and terms, Table S2: Extraction form.
