# Supplementary material for: An Evidence-Based Rapid Review of Surgical Techniques for Correction of Prolapsed Nictitans Glands in Dogs
Source: Vet Sci. 2018 Aug 23;5(3):75. doi: 10.3390/vetsci5030075 (PMC6163435; doi:10.3390/vetsci5030075)
Supplement: Supplementary file 1 [file vetsci-05-00075-s001.pdf]

Supplementary Materials: An Evidence-Based Review of Surgical Techniques for Correction of Prolapsed Nictitans Glands in Dogs

Constance White and Marnie L. Brennan *

Table S1. Search strategies and terms.

Database	Search
**CAB Abstracts (1910-present) (Ovid interface)**	(canine or canines or canid or canids or canis or canidae or dog or dogs).mp. or exp Dogs/ or exp Canidae/<br>**AND**<br>(nictitan or nictitans or third eyelid or third eyelids or cherry eye or cherry eyes or nictitating membrane or nictitating membranes).mp.<br>**AND**<br>(prolapse or prolapsed or prolapses or replacement or replacements or replaced or replace or surgery or surgical or repair or amputate or amputated).mp. or exp prolapse/ or exp surgery/
**Medline (In-Process & Other Non-Indexed Citations and MEDLINE(R) 1946 to Present (Ovid interface)**	(canine or canines or canid or canids or canis or canidae or dog or dogs).mp. or exp Dogs/ or exp Canidae/<br>**AND**<br>(nictitan or nictitans or third eyelid or third eyelids or cherry eye or cherry eyes or nictitating membrane or nictitating membranes).mp. or exp Nictitating Membrane/<br>**AND**<br>(prolapse or prolapsed or prolapses or replacement or replacements or replaced or replace or surgery or surgical or repair or amputate or amputated).mp. or exp Prolapse/ or exp General surgery/
**PubMed (native interface)**	(((((((((((canine) OR canines) OR canis) OR canis) OR canis) OR canidae) OR dog) OR dogs) OR Dogs[MeSH Major Topic]) OR Canidae[MeSH Major Topic])) AND ((((((((((nictitans) OR nictitans) OR third eyelid) OR third eyelids) OR cherry eye) OR cherry eyes) OR nictitating membrane) OR nictitating membranes) OR nictitating membrane[MeSH Major Topic])) AND (((((((((((((((prolapse) OR prolapse) OR prolapses) OR replacement) OR replacements) OR replaced) OR replace) OR surgery) OR surgical) OR repair) OR amputated) OR amputated) OR prolapse[MeSH Major Topic]) OR surgery[MeSH Major Topic]))

Table S2. Extraction form.

Publication		
Variable	Reported	Details
dogs/eyes	**R** all dogs and eyes enumerated<br>**PR** dogs enumerated or eyes enumerated but not both<br>**NR** neither dogs nor eyes enumerated	
Procedure		
Reported design	**R** Authors presented key elements of study design early in the paper<br>**NR** Authors did not describe study type or present key elements of study design early in the paper	
Study type	**R** Author indicated type of study design and key elements<br>**NR** Authors did not indicate study design and key elements	1. Experimental<br>Randomized interventions (parallel or crossover)<br>2. Observational Population Cohort<br>a. Enrolled as a result of exposure (PNG with more than one **intervention group**), incidence or enumeration of outcomes (recurrence and/or lacrimal results) reported<br>3. Surgical Case Series<br>Cases enrolled as a result of exposure (PNG with **single intervention group**) with incidence or enumeration of outcomes (recurrence and/or lacrimal results) reported. (Some authors suggest that this may also be classified as a descriptive cohort study).
Case selection (sampling method)	**R** Reported source, eligibility criteria and methods of selection of cases<br>**PR** Reported one or two of the above<br>**NR** Did not report any of the above	
Follow-up time	**R** Reported follow-up time for all reported outcomes on all patients<br>**PR** Reported follow-up time for some but not all outcomes, or some but not all patients<br>**NR** Did not report follow-up time for any outcome	
Follow-up method		
Loss to follow-up	**R** Numbers of cases at each stage of study enumerated and/or patient-level follow-up times reported; reasons for non-participation given; explanation of how missing data and/or loss to follow-up was addressed in statistical analysis (if statistical analysis performed)<br>**PR** Number of cases at each stage of the study enumerated	

	**Unclear** Cohort inclusion may have depended on minimum follow-up period/availability of case records
Prolapse duration	**R** Prolapse duration prior to surgery reported either as summary statistic, range, or patient-level data<br>**NR** No data given on prolapse duration
Patient demographics enrollment	**R** Breeds enumerated for all patients in study. Ages given as summary statistic and/or at patient level<br>**PR** Reporting of the above for part, but not all, of the cohort; or only breed or age but not both given.<br>**NR** No demographics provided for any of cohort
Patient demographics outcomes	**R** Surgical failure or lacrimal outcomes reported and/or analyzed by breed<br>**PR** Outcomes adjusted for or reported for selected breeds but not all breeds in cohort<br>**NR** Surgical failure or lacrimal outcomes not reported for and/or analyzed by breed
Pre-operative steroid	**R** Pre-operative care reported in sufficient detail to know which patients, if any, received pre-operative topical steroid<br>**PR** Pre-operative care reported in insufficient detail to know which patients, if any, received pre-operative topical steroid<br>**NR** Pre-operative care not reported sufficiently to know if any patients received pre-operative steroid
Postop steroid	**R** Post-operative care reported in sufficient detail to know which patients, if any, received post-operative topical steroid<br>**PR** Post-operative care reported in insufficient detail to know which patients, if any, received post-operative topical steroid<br>**NR** Post-operative care not reported sufficiently to know if any patients received post-operative steroid
Recurrence	**R** Reprolapse after surgical correction reported in sufficient detail to present or calculate a surgical failure rate per eye<br>**PR** Reprolapse after surgical correction reported in sufficient detail to present or calculate a surgical failure rate per dog but not per eye<br>**NA** No possibility of recurred prolapse (e.g. excision)
Time to recurrence	**R** reported summary statistic or patient-level data on time to surgical failure

	**PR** Time to recurrence provided for some but not all cases of surgical failure<br>**NR** No data on time to surgical failure<br>**NA** No recurrences possible (excision) or observed
Complications	Post-operative complications aside from recurrence
STT	**R** Post-operative STT results reported for some or all cases<br>**NR** No STT data presented for any cases
KCS	**R** Report incidence or number of post-operative KCS for all included cases<br>**PR** Reporting of post-operative incidence for subset of cases<br>**NR** No reporting of post-operative KCS incidence
KCS criteria	**R** Criteria for diagnosis of KCS defined<br>**NR** No criteria given for diagnosis of KCS<br>**NA** KCS not reported

© 2018 by the authors. Submitted for possible open access publication under the terms and conditions of the Creative Commons Attribution (CC BY) license (http://creativecommons.org/licenses/by/4.0/).
